# PHL1/341: Guidelines for Designing Education Resources for the World Wide Web: Strategies from the Field

**DOI:** 10.2196/jmir.1.suppl1.e82

**Published:** 1999-09-19

**Authors:** S Koyani, N Beidler

**Affiliations:** ^1^National Cancer InstituteBethesdaUSA

**Keywords:** Internet, Health Education, Guidelines, Design, Publications

## Abstract

**Introduction:**

An increasing number of professionals, patients, and members of the general public are using the World Wide Web to obtain and/or deliver health information. Although the Web can serve as a highly effective medium for educating and informing, little information is available about how to design effective, accessible, and readable online health information. Additionally, few, if any, comprehensive standards exist on designing educational materials for a Web environment. In response, the National Cancer Institute's (NCI) Office of Cancer Information, Communication, and Education (OCICE) developed guidelines on how to design readable, intuitive, and easy-to-navigate health information for patients, the general public and health professionals. Using the guidelines as a framework, this presentation will include:

- guidance in identifying whether a resource is appropriate for the Web
- design principles and technical considerations when developing online resources
- demonstration of NCI publications designed for online viewing
- examples of good and bad design
- suggestions for implementing the guidelines in academic, community, and clinical settings

**Methods:**

The NCI Guidelines for Designing Educational Resources for the WWW are based on an extensive review of the literature for design, cognitive learning, and Web usability data; reviews of popular health Web sites; Web usability tests conducted by NCI with patients and health professionals; and discussions with experts in the field.

**Results:**

Research data was used to develop the guidelines document, as well as online models of NCI education resources for cancer patients and the public. Initial results indicate improved readability and satisfaction. A plan will be set in place to design all NCI print and online education publications using these guidelines. Additionally, the guidelines will be updated on a continual basis following further testing and evaluation.

**Discussion:**

Since the Internet is increasingly being used as a health information delivery and retrieval vehicle, it is imperative that information is optimally designed and presented to the user. Although the NCI guidelines are a starting point, more research is needed to identify changing user needs and trends, new delivery software, and strategies to tailor information based on the users' preference. The guidelines will continue to be refined and updated as new research and testing results become available.

